# Supplementary material for: In-layer inhomogeneity of molecular dynamics in quasi-liquid layers of ice
Source: Commun Chem. 2024 May 29;7:117. doi: 10.1038/s42004-024-01197-0 (PMC11136980; doi:10.1038/s42004-024-01197-0)
Supplement: Supplementary file 2 — SUPPLEMENTAL Information [file 42004_2024_1197_MOESM2_ESM.pdf]

Supplementary Information for "In-layer inhomogeneity of molecular dynamics in quasi-liquid layers of ice"

Ikki Yasuda,[1] Katsuhiro Endo,[1] Norityoshi Arai,[1] and Kenji Yasuoka*,[1]

[1] *Department of Mechanical Engineering, Keio University, Yokohama, Japan*

*Corresponding author. E-mail: yasuoka@mech.keio.ac.jp

SUPPLEMENTARY METHOD

Unsupervised deep learning

Local dynamics ensembles [1], that is, the probability distributions of short-term trajectories, were assumed for the solid and the combined systems of the solid and water. The difference between the two local dynamics ensembles was measured based on the Wasserstein distance W [2, 3]. For high-dimensional distributions, the Wasserstein distance cannot be solved using linear programming due to the dimensionality. Rather, the optimal transport function is approximated using a deep neural network,

$$W = \sup_{||f||_{L \leq 1}} \mathbb{E}_{\boldsymbol{x} \sim \boldsymbol{y}} \left[f(\boldsymbol{x}) \right] - \mathbb{E}_{\boldsymbol{x}' \sim \boldsymbol{y}'} \left[f(\boldsymbol{x}') \right] \tag{1}$$

where $\boldsymbol{x}$ and $\boldsymbol{x}'$ are short-term trajectories for the concatenated and solid systems, respectively. The function f^* is the 1-Lipschitz function expressed by a deep learning model that is identical as used in our previous work [1], i.e. four layers of dense neural network are built as input(1024)–hidden(1024)–hidden(1024)–hidden(1024)–output(1), where the number inside () is the number of output nodes. To restrain the 1-Lipschitz constraint, the gradient penalty term was added to the loss function [4]. The model was optimized for 100,000 steps using Adam optimizer [5]. To make the output of the neural network stable, we used a large minibatch size, i.e. 2048, for the training and the calculation of $g(\boldsymbol{x})$. The algorithm to calculate $g(\boldsymbol{x})$ is described in the Supplementary information of ref [6].

---

[1] K. Endo, D. Yuhara, K. Tomobe, and K. Yasuoka, Detection of molecular behavior that characterizes systems using a deep learning approach, Nanoscale **11**, 10064 (2019).

[2] C. Villani, *Optimal transport: old and new*, Vol. 338 (Springer Science & Business Media, 2008).

[3] M. Arjovsky, S. Chintala, and L. Bottou, Wasserstein generative adversarial networks, in *Proceedings of the 34th International Conference on Machine Learning*, Vol. 70 (2017) pp. 214–223.

[4] I. Gulrajani, F. Ahmed, M. Arjovsky, V. Dumoulin, and A. Courville, Improved training of wasserstein gans, in *Advances in neural information processing systems*, Vol. 30 (2017) pp. 5767–5777.

Supplementary Software 1 Pesudo-code to calculate $g(x)$. LDE of system i is denoted as y_i.

Require: : f^*, Trained neural network to calculate Wasserstein distance between system i and j. N_i, Number of molecules in system i. T_i, Number of MD frames in system i. m, The batch size. Δt, time of LDE. δt, interval time step of MD frame used in LDE.

for $n = 1, ..., N_i$ **do**
  for $t = 1, ..., T_i$ **do**
    Sample x_i the short-term trajectory in system i of molecular index n at frame t to $t+\Delta t$ at interval δt.
    Sample $\{x_j\}_{k=1}^{m} \sim y_j$ short-term trajectories from system j.
    $g(x_i) \leftarrow f^*(x_i) - \frac{1}{m}\sum_{k=1}^{m} f^*(x_j)$
  end for
end for
for $n = 1, ..., N_j$ **do**
  for $t = 1, ..., T_j$ **do**
    Sample x_j the short-term trajectory in system j of molecular index n at frame t to $t+\Delta t$ at interval δt.
    Sample $\{x_i\}_{k=1}^{m} \sim y_i$ short-term trajectories from system i.
    $g(x_j) \leftarrow -f^*(x_j) + \frac{1}{m}\sum_{k=1}^{m} f^*(x_i)$
  end for
end for

[5] D. P. Kingma and J. Ba, Adam: A method for stochastic optimization, arXiv preprint arXiv:1412.6980 (2014).

[6] I. Yasuda, K. Endo, E. Yamamoto, Y. Hirano, and K. Yasuoka, Differences in ligand-induced protein dynamics extracted from an unsupervised deep learning approach correlate with protein–ligand binding affinities, Commun. Biol. **5**, 481 (2022).

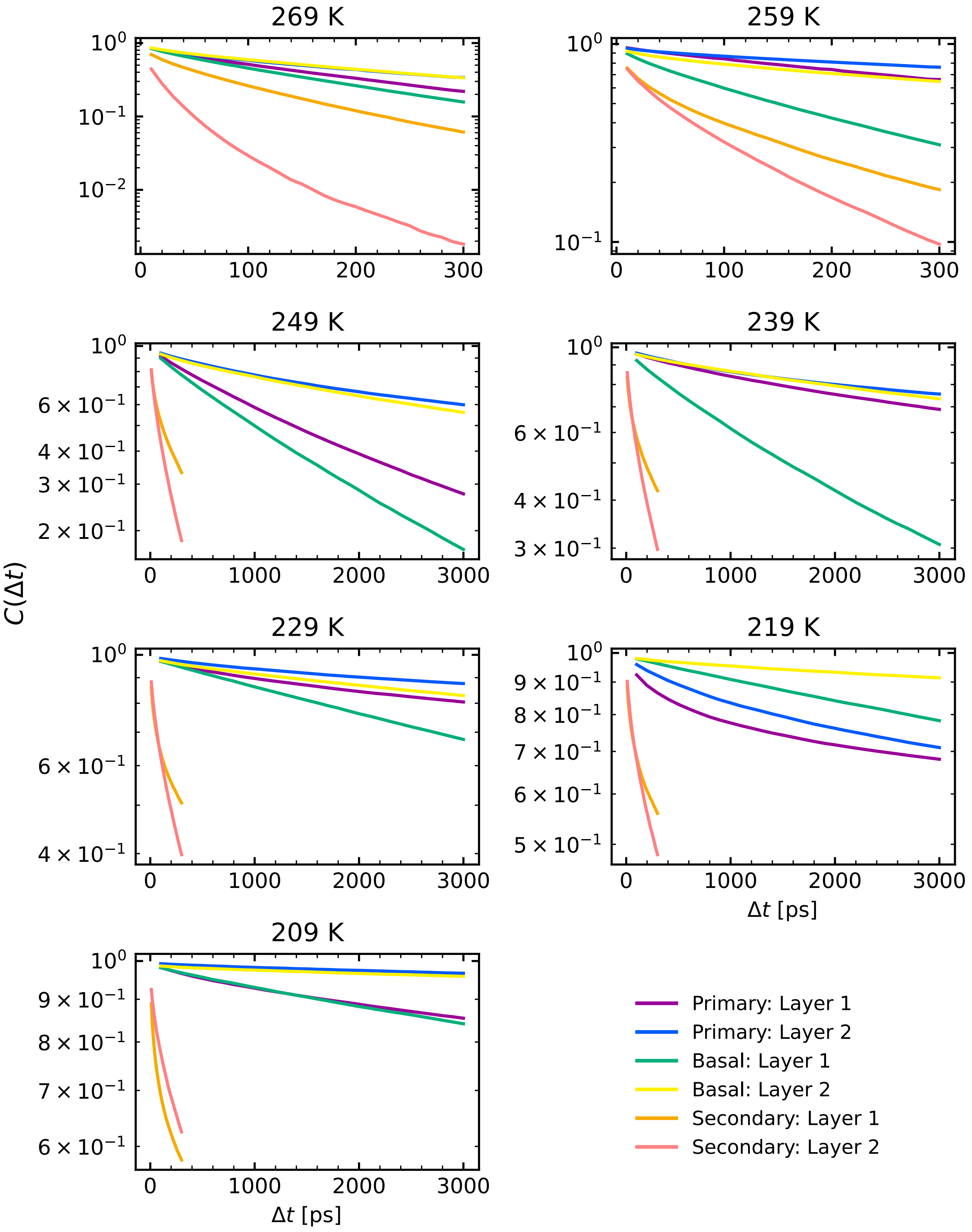

Supplementary Figure 1. Survival time correlation $C(\Delta t)$ for molecules to stay in a layer.

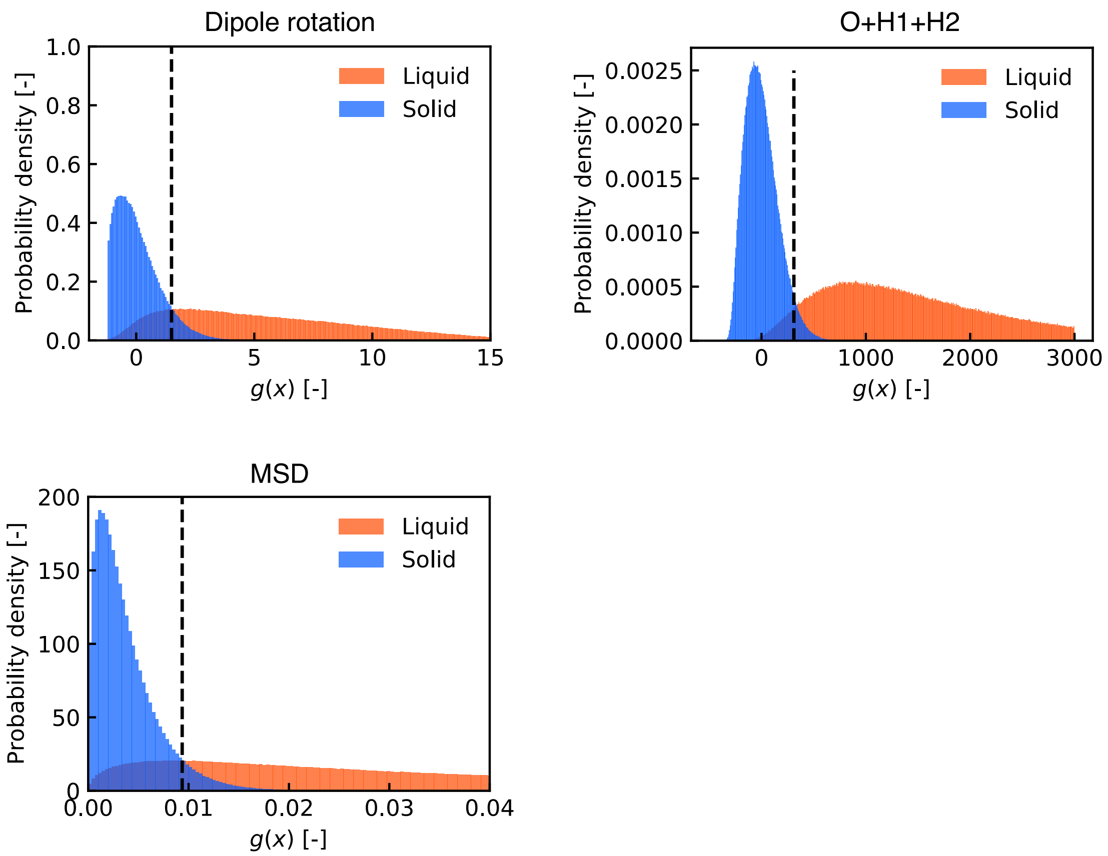

Supplementary Figure 2. Profiles of $g(\boldsymbol{x})$ using different types of dynamics as input. Time length of local dynamics ensemble is 64 ps. Black dashed line shows the classification boundary.

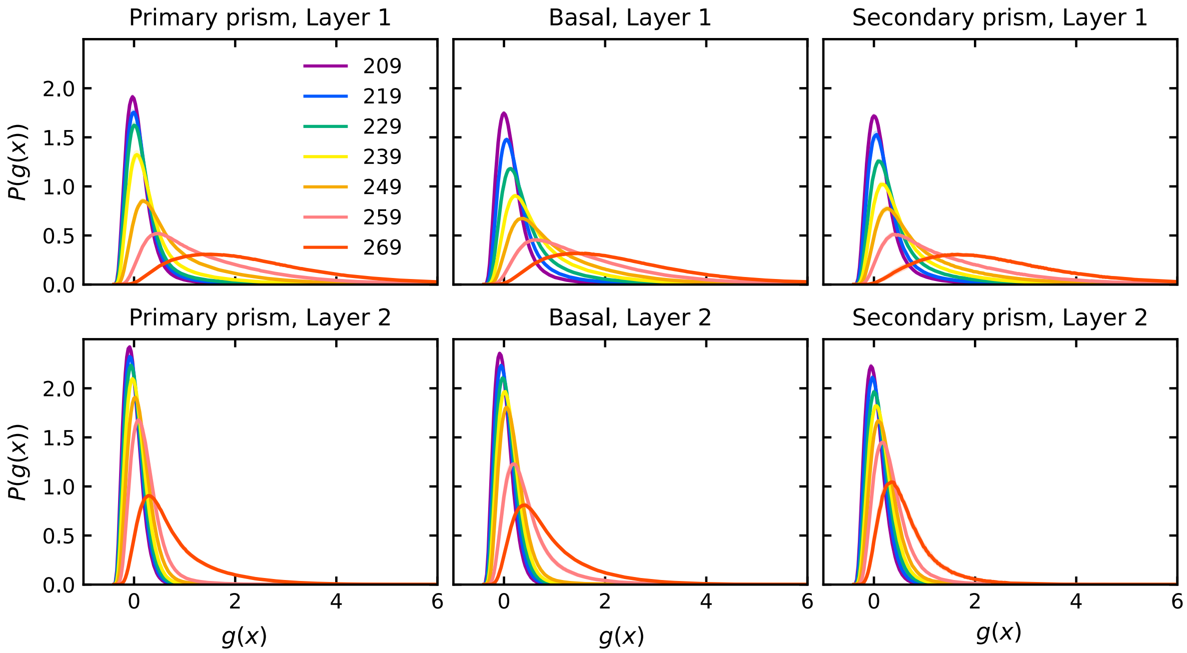

Supplementary Figure 3. Profile of $g(\boldsymbol{x})$ in Layer 1 and 2 at different faces and temperature.

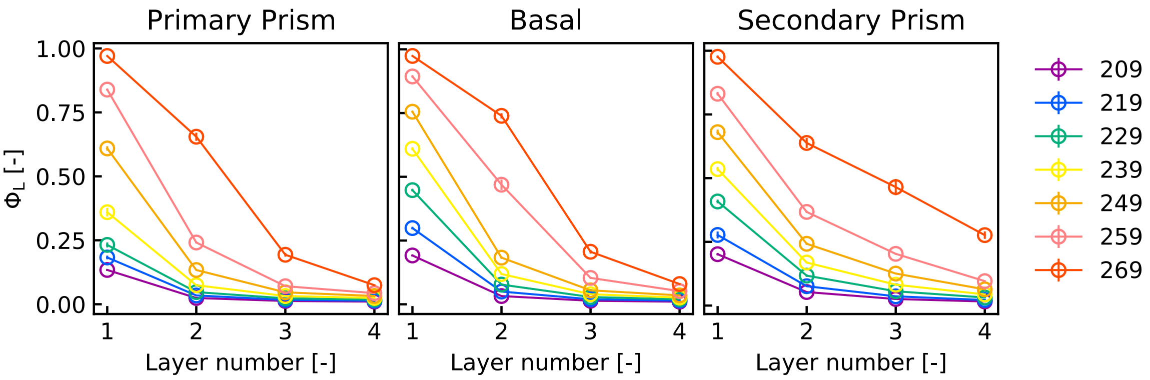

Supplementary Figure 4. Liquid ratio Φ_L in Layer 1–4 at different faces and temperature.
