# Supplementary material for: In-layer inhomogeneity of molecular dynamics in quasi-liquid layers of ice
Source: Commun Chem. 2024 May 29;7:117. doi: 10.1038/s42004-024-01197-0 (PMC11136980; doi:10.1038/s42004-024-01197-0)
Supplement: Supplementary file 3 — Description of Additional Supplementary Files [file 42004_2024_1197_MOESM3_ESM.pdf]

Description of Additional Supplementary Files

File name- Supplementary Movie 1

File description-In Data availability section, a sentence is added as “Simulation movie of basal face of Layer 2 at 259 K is available in the file Supplementary Movie 1.
